# Refinement of a radioreceptor binding assay for nicotinic acid adenine dinucleotide phosphate

**DOI:** 10.1016/j.ab.2007.08.030

**Published:** 2007-12-01

**Authors:** Alexander M. Lewis, Roser Masgrau, Sridhar R. Vasudevan, Michiko Yamasaki, John S. O’Neill, Clive Garnham, Kristin James, Andrew Macdonald, Mathias Ziegler, Antony Galione, Grant C. Churchill

**Affiliations:** a Department of Pharmacology, University of Oxford, Oxford OX1 3QT, UK; b Department of Molecular Biology, University of Bergen, N-5008 Bergen, Norway

**Keywords:** NAADP, Mammalian, Second messenger, Radioreceptor

## Abstract

The measurement of changes in nicotinic acid adenine dinucleotide phosphate (NAADP) levels in cells has been, and remains, key to the investigation of the functions of NAADP as a Ca^2+^-releasing second messenger. Here we provide details of how to isolate NAADP from cells by extraction with perchloric acid and then measure the NAADP using a radioreceptor assay. We demonstrate that NAADP is neither generated nor broken down during sample processing conditions and that radioreceptor assay is highly selective for the detection of NAADP under cell extract conditions. Furthermore, a number of improvements, such as solid-state detection of the radioactivity, are incorporated to enhance the safety of the procedure. Finally, we have developed a new method to prevent the endogenous metabolism of NAADP by chelating Ca^2+^ with bis-(*o*-aminophenoxy)ethane-*N,N,N′,N′*-tetraacetic acid (BAPTA), thereby reducing the difficulty of catching a small transient rise in NAADP levels. In summary, we have refined and improved a method for measuring NAADP levels and presented it in a manner accessible to a wide range of laboratories. It is expected that this will enhance research in the NAADP field.

The cytosolic concentration of Ca^2+^ is very closely regulated in cells; the resting concentration usually is maintained at approximately 10^–7^ M. Changes in Ca^2+^ concentration play an important role in a wide range of processes—from fertilization, to the regulation of cell function throughout life, to cell death [1]. This resting concentration may be increased by means of influx of Ca^2+^ from the extracellular matrix through a channel in the plasma membrane or release of Ca^2+^ from intracellular stores. Release of Ca^2+^ from intracellular stores usually is a result of the binding of a primary messenger, such as a hormone or neurotransmitter, to a receptor in the plasma membrane. This in turn results in the generation of a second messenger inside the cell that is able to release Ca^2+^ from an intracellular store. The best-characterized Ca^2+^-releasing second messenger is d-myo-inositol 1,4,5-trisphosphate (IP_3_).^6^ It is synthesized following the activation of a receptor that is coupled to G_q_, which in turn activates phospholipase C to cleave IP_3_ from phosphatidylinositol 4,5-bisphosphate. IP_3_ binds to the IP_3_ receptor in the endoplasmic reticulum membrane and releases Ca^2+^ from the endoplasmic reticulum [2].

Nicotinic acid adenine dinucleotide phosphate (NAADP) was first reported as a Ca^2+^-releasing agent in 1995 [3]. However, in contrast to IP_3_, very little is known about the physiological roles of NAADP. The site of action of NAADP remains controversial; there is evidence to suggest that NAADP releases from an acidic lysosome-like store [4,5], although there are also reports of it affecting ryanodine receptors on the endoplasmic reticulum [6]. NAADP has now been categorized as a second messenger, according to the conditions laid down by Sutherland and coworkers [7], in both the sea urchin [8] and model mammalian [9] systems. The ability to measure changes in NAADP levels in cells has been key to this categorization, providing evidence that NAADP is synthesized in response to the binding of an agonist to its cell surface receptor. This remains very important in characterizing NAADP in a wider range of cell types, enabling a more comprehensive picture of the functions of NAADP as a second messenger to be built up. Basal NAADP levels have been measured in a variety of sea urchin [10], plant [11], and mammalian [12,13] cell types. Only recently, however, have agonist-induced changes in NAADP levels been reported in sea urchin sperm [8], pancreatic beta cells [9], smooth muscle cells [14], pancreatic acinar cells [15], and T cells [13]. The majority of these measurements have been made using a radioreceptor binding assay [12], although a cycling assay [13,16,17] has also been described.

In theory, it is possible to use a bioassay in sea urchin egg homogenate, similar to that used for cyclic ADP ribose (cADPR) [18], to detect NAADP. However, there are no reports of NAADP measurements using such a technique. Three variations on a cycling assay have been reported [13,16,17]. These assays offer sensitive detection of NAADP, with 50-fmol quantities being detectable with one method [13], and have the benefit that they do not require the preparation of a radioligand. However, the processing of samples is technically demanding and time-consuming. Cell extracts require purification by anion exchange chromatography and subsequent enzyme treatment prior to determination of the NAADP concentration, which is actually determined indirectly from the resulting concentration of nicotinamide adenine dinucleotide (NAD) or nicotinamide adenine dinucleotide phosphate (NADP). The radioreceptor binding assay that we report here is capable of directly detecting 100-amol quantities of NAADP without the risk of interference from other compounds. Furthermore, it is possible to quickly process a large number of samples prepared using a straightforward acid extraction technique without the need for further purification.

Previously expressed concerns encompassing the generation or degradation of NAADP, as well as the selectivity of the assay, and the recovery of NAADP in a cell extract are addressed in detail. Importantly, we report a new method where NAADP increases are measured as NAADP accumulation over time. In summary, we have refined and validated a powerful method for measuring NAADP levels, and we present it in an accessible manner that will enable even inexperienced researchers to measure NAADP levels. The importance of these measurements at this stage in our understanding of the functions of NAADP cannot be overlooked. It is expected that this will enhance research in the NAADP field.

## Materials and methods

### Materials

All chemicals were purchased from Sigma (UK) except where otherwise indicated below.

### High-performance liquid chromatography

All high-performance liquid chromatography (HPLC) was carried out on an anion exchange resin (AGMP1, Bio-Rad, USA), packed in 150 × 2.5-mm Omnifit columns, using a concave upward gradient of trifluoroacetic acid (TFA), delivered by a Waters 600e pump, as described previously [19]. Detection was performed by a UV detector (Waters 2487, UK) at 254 nm.

### Stability of nucleotides under different pH conditions

NADP, NAADP, or NADPH (reduced-form NADP) (1 mM) was incubated in the following buffers for 6 h: 50 mM Hepes (pH 7.2), 0.75 M HClO_4_ (pH 0.88), 1 M KHCO_3_ (pH 9.1), 0.75 M HClO_4_/1 M KHCO_3_ (pH 8.6), or 1 M K_2_CO_3_ (pH 11). Samples were then neutralized with their base equivalence of either Tris base (for acidic solutions) or Hepes acid (for basic solutions) as applicable. Samples were analyzed by HPLC as described above, with elution times being determined from standard runs with known compounds.

### Acid extraction of NAADP

Cells may be prepared in any suitable manner; gametes, isolated cells, chopped tissue, and cells in culture all have been used successfully. Cells were then exposed to agonist, and at the appropriate time points the reaction in cells was stopped by the addition of 0.75 M ice-cold HClO_4_ added as an equal volume of 1.5 M solution. Sonication (Jencons Vibracell at amplitude 60) was carried out to disrupt the cells, which were then placed on ice for 15 min. The denatured protein was pelleted by centrifugation at 9000*g* for 10 min and stored at –80 °C for later analysis. Supernatant was neutralized with 1 M KHCO_3_, added as an equal volume of 2 M solution, and vortexed. The resulting KClO_4_ precipitate was removed by centifugation at 9000*g* for 10 min. Samples were stored at –80 °C for later analysis.

### Determination of percentage recovery of NAADP by acid extraction

*Lytechinus pictus* (sea urchins) were given intracoelomic injections of 1 ml of 0.5 M KCl. Eggs were harvested into artificial sea water (ASW) with the following composition: 435 mM NaCl, 15 mM MgSO_4_, 11 mM CaCl_2_, 10 mM KCl, 2.5 mM NaHCO_3_, and 20 mM Tris–Cl (pH 8.0). Sperm was collected “dry” on a Petri dish. Samples consisted of 20 μl sperm and 80 μl eggs in ASW. The samples were spiked with [^32^P]NAADP and then processed as described later. The resulting solution was then counted, and the total percentage recovery was determined.

### Isolation and use of pancreatic acinar cells

Pancreatic acinar cells were prepared as described previously [15]. Briefly, pancreata were excised from 8- to 10-week-old male CD1 mice and were finely chopped before incubation with 200 U/ml collagenase for 15 min at 37 °C. The digested tissue was then washed and resuspended to 10 ml with buffer of the following composition: 140 mM NaCl, 4.7 mM KCl, 1 mM MgCl_2_, 1 mM CaCl_2_, 10 mM Hepes, and 10 mM glucose (pH 7.4). Protease inhibitor cocktail tablets (EDTA free, Roche Diagnostics, UK), one tablet per 50 ml, were added to this solution before use. The suspension was triturated, and supernatant that contained dispersed cells was collected into a new tube. After centrifugation at 800*g* for 3 min at 4 °C, the supernatant was discarded and the cell pellet was resuspended for further trituration and centrifugation. The resulting supernatant was discarded, and the cells were kept in buffer supplemented with 1 mM CaCl_2_ at room temperature on a rocking board for 30 min before use. Prior to use, the cells were centrifuged at 800*g* for 1 min at 4 °C. The cells were suspended in the above buffer without the addition of protease inhibitors. Pancreatic acinar cells were aliquoted into tubes. These aliquots were incubated with 20 μM 1,2-bis(2-aminophenoxy)ethane-*N,N,N′,N′*-tetraacetic acid tetrakis (acetoxymethyl) ester (BAPTA-AM), a cell-permeant Ca^2+^ chelator, for 30 min at room temperature. Cholecystokinin (CCK), or buffer control, was then added to each aliquot by pipette. Further sample preparation was carried out as described earlier.

### [^32^P]NAADP synthesis

[^32^P]NAADP was synthesized in a two-step reaction as described previously [18,20]. First, [^32^P]NADP was synthesized by incubating 25 μl/9.25 MBq [^32^P]NAD (GE Healthcare, UK) with 0.5 U/ml human NAD kinase (formerly kindly provided by Matthias Ziegler, currently obtained commercially from Alexis, UK), 5 mM MgATP, and 100 mM Hepes (pH 7.4) for 1 h. Then 100 mM nicotinic acid and 1 μg/ml ADP-ribosyl cyclase (kindly provided by H. C. Lee, University of Hong Kong) were added to commence the second step, which was allowed to proceed for 1 h. The resulting mixture was pumped onto an HPLC column with a peristaltic pump. Separation was carried out on an anion exchange resin (AGMP1) using a concave upward gradient of TFA as described previously [20–23] with detection using an in-line Geiger counter (Bioscan Flow Count B-FC-1000, Bioscan, USA) integrated to the system using a Waters bus SAT/IN unit. The NAADP fraction was then collected and stored in 10 mM Tris at 4 °C for use in the assay. The 14-day half-life of ^32^P limits use of the [^32^P]NAADP to approximately 6 weeks from synthesis.

### Sea urchin homogenate preparation

Sea urchin egg homogenate was prepared as described previously [18]. Briefly, eggs were harvested from *L. pictus* by intracoelomic injection of approximately 1 ml of 0.5 M KCl. Homogenate was then prepared as described previously by Dargie and coworkers [24]. Briefly, the eggs were washed several times by centrifugation at 800*g* in the following solutions: Ca^2+^-free ASW with EGTA (twice), Ca^2+^-free ASW (twice), and “intracellular medium.” The intracellular medium had the following composition: 250 mM *N*-methyl-d-glucamine, 250 mM potassium gluconate, 20 mM Hepes, and 1 mM MgCl_2_ (pH adjusted to 7.2 with acetic acid). Homogenization was performed in intracellular medium, to which the following were added with a Dounce glass tissue homogenizer with a size A pestle: 2 mM ATP, 20 mM phosphocreatine, 20 U/ml creatine phosphokinase, 50 μg/ml leupeptin (EDTA-free cocktail), 20 μg/ml aprotinin, and 100 μg/ml soya bean trypsin inhibitor. Cortical granules were removed by centrifugation at 13,000*g* for 10 s at 4 °C. Homogenate was stored in 0.5- to 1-ml aliquots at –80 °C for up to 5 years and were used immediately after thawing.

### NAADP binding

NAADP binding protein from sea urchin (*L. pictus*) egg homogenate, which is highly selective for NAADP [20,23,25], was used. First, 25 μl of test sample was added to each tube. Second, 125 μl of 1% (v/v) sea urchin egg homogenate in intracellular medium was added and allowed to incubate for 10 min at 25 °C. Finally, 100 μl of [^32^P]NAADP diluted into intracellular medium was then added to generate approximately 50,000 scintillation counts per tube. The tubes were then incubated at room temperature for a further 10 min at 25 °C. Note that the order of addition is important to maximize the sensitivity of the assay [12]. Bound NAADP was then trapped onto Whatman GF/B filter papers using a Brandel Cell Harvester. Washing was carried out three times using 1 ml of a buffer containing 20 mM Hepes and 500 mM potassium acetate (pH 7.4).

### Scintillation counting

Radioactivity was determined by placing the filter circles in a scintillation vial containing 10 ml H_2_O, followed by Cerenkov scintillation counting.

### Storage phosphor detection

Filter papers were wrapped in cling film and then placed in a cassette to expose a storage phosphor screen (GE Healthcare) for 1 to 4 h. The screen was then scanned using a Typhoon 9400 scanner (GE Healthcare) at a resolution of 100 μm. The resulting image was then analyzed using ImageQuant (GE Healthcare).

## Results and discussion

**Fig. 1:**
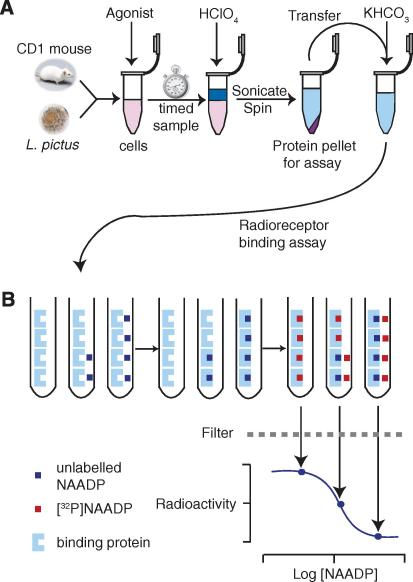
(A) Schematic diagram of the acid extraction process for NAADP. Sperm or eggs are harvested from *L. pictus,* or cells are prepared from male CD1 mouse pancreas. Agonist is incubated with the cell preparation, and then HClO_4_ is added to stop the reaction at the required time point. The sample is then sonicated to disrupt the cells and is centrifuged to pellet the protein for subsequent assay. The supernatant is neutralized with an equal volume of 2 M KHCO_3_, or other bases where indicated, in preparation for analysis using the radioreceptor binding assay. (B) Schematic diagram of the NAADP radioreceptor binding assay. First, known concentrations of NAADP (blue boxes), or cell extracts, are added, followed by sea urchin egg homogenate (pale blue shapes) in intracellular medium and a 10-min incubation period. NAADP binds irreversibly to the receptors in the homogenate. [^32^P]NAADP (red boxes) is then added. This binds to the remaining available receptor sites. Bound NAADP is separated from the mixture by filtration, and the radioactivity is determined. Sample NAADP concentrations may be determined from the standard curve. (For interpretation of the references to color in this figure legend, the reader is referred to the Web version of this article.)

Measurement of NAADP levels, both at rest and in response to agonist stimulation, has been central to the initial demonstration of NAADP as a Ca^2+^-releasing second messenger. It is now becoming an ever more important process as the roles of NAADP are investigated in more systems and the agonists that are coupled to increases in NAADP levels are fully defined. The measurement of NAADP in cells may be divided into two steps. First, the NAADP is isolated from the cells by means of acid extraction (Fig. 1A). Second, the NAADP in these samples is measured using the radioreceptor binding assay (Fig. 1B). In the first step, cells are isolated from the relevant animal; here we have used pancreatic acinar cells from CD1 mice and sea urchin (*L. pictus*) eggs and sperm. They are then exposed to agonist, and the reaction is stopped at the appropriate time point by the addition of HClO_4_. Cultured cells may be used as an alternative to a freshly prepared cell suspension; in this case, they should be scraped off the tissue culture plate at this stage. Sonication is used to disrupt the cells, and the protein is then pelleted. The resulting supernatant is neutralized with KHCO_3_ prior to analysis by the radioreceptor binding assay. Therefore, it is necessary to establish that this acid extraction is indeed a valid method for isolation of NAADP from cells and to determine how much NAADP is recovered using this method. The radioreceptor binding assay itself is described and validated in detail. Finally, a number of improvements to the process are described.

### Stability of NAADP during acid extraction process

During the acid extraction process, HClO_4_ is added to the cells, producing an acidic pH (0.88), and this solution is later neutralized, producing an alkaline pH (8.6). Therefore, it must be confirmed that NAADP is stable under the conditions used and that no other nucleotides may be converted to NAADP under these conditions. It is commonly stated that in their oxidized form NAD and NADP are stable in acidic solutions but unstable in alkaline ones [26]. In contrast, their reduced forms, NADH and NADPH, are stable in alkaline solutions but unstable in acidic ones [26]. In addition, alkaline treatment of NADP has been used to convert it to NAADP [27]. Moreover, Churamani and coworkers [12] reported that acid extraction using trichloroacetic acid caused conversion of NADP to NAADP. Therefore, we investigated the effect of our extraction conditions on the stability of NADP, NADPH, and NAADP.

**Fig. 2:**
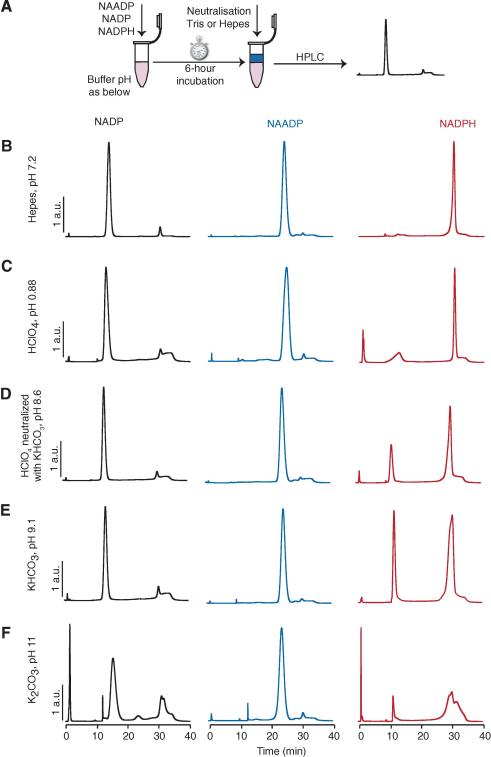
Stability of nucleotides during the acid extraction procedure. (A) Schematic showing the procedure. NAADP, NADP, or NADPH (1 mM) was incubated for 6 h in the solutions indicated. Samples were then neutralized by the addition of Hepes acid for basic samples, or Tris base for acidic samples, and were analyzed by HPLC. HPLC traces show the stability of NAADP, NADP, and NADPH in Hepes (pH 7.2) (B), HClO_4_ (pH 0.88) (C), HClO_4_ neutralized with KHCO_3_ (pH 8.6) (D), KHCO_3_ (pH 9.1) (E), and K_2_CO_3_ (pH 11) (F). HPLC traces are scaled to a common peak height for comparison (maximum 20% scaling). Note that NADP is stable except at pH 11, when a number of products result; in particular, NAADP is generated, and this would interfere with correct determination of NAADP levels from a cell extract. NADPH is stable only at pH 7.2, but NADP is the principal breakdown product; NAADP is not produced under the conditions tested. a.u., arbitrary unit.

NADP, NAADP, and NADPH were incubated for 6 h in solutions with a range of pH (0.88–11). Where necessary, these solutions were neutralized with their base equivalence of Hepes acid or Tris base prior to analysis by anion exchange HPLC (Fig. 2A). NADP, NAADP, and NADPH remained stable at pH 7.2 (Fig. 2B). However, at pH 0.88, 8.6 and 9.1 NADPH became unstable, whereas NADP and NAADP remained stable (Figs. 2C–E). NADPH broke down to NADP (15 min) and uncharged products (0–2 min); these would not interfere with the detection of NAADP and, hence, were not considered to be a problem. At pH 11, NADPH proved to be similarly unstable; some conversion of NAADP to NADP occurred. More worrisome, NADP converted to a variety of products, including NAADP (25 min) (Fig. 2F). Therefore, it was decided to thoroughly investigate the range of pH that might be encountered under the extraction conditions of the assay, with particular reference to the possibility of overshooting the intended pH when neutralizing with base.

### Titration of HClO_4_ with different bases

**Supplementary Figure 1:**
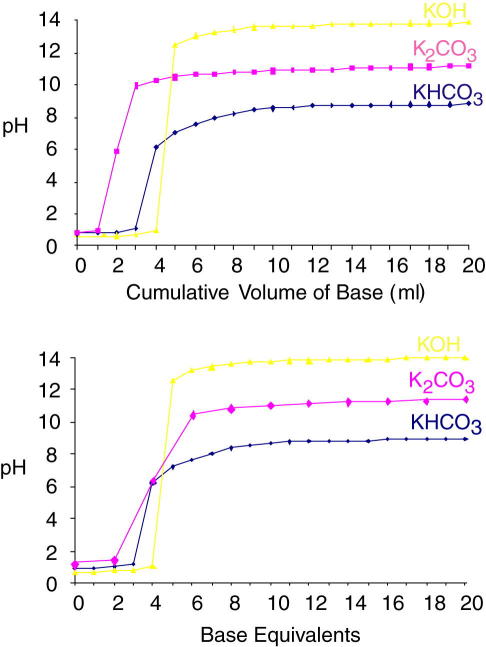


HClO_4_ is a strong monoprotic acid, and a 0.75 M solution has a pH of approximately 0.7. There are several choices of potassium bases that can be used for neutralization, producing a precipitate of KHClO_4_. Given the sensitivity of both NADP and NAADP to alkaline pH, we investigated a range of these possibilities. Titration of 1.5 M HClO_4_ with 1 M KOH, which has been reported as a neutralizing base [28], gave a sharp equivalence point and resulted in a maximum pH of between 13 and 14 (see Supplementary Fig. 1). The sharp equivalence point makes it difficult to add precisely the correct amount to neutralize the solution without inadvertently generating an alkaline pH. Therefore, this base was considered to be unsuitable for use in this technique. K_2_CO_3_ may also be used for neutralizations. When 1.5 M HClO_4_ was titrated with 2 M K_2_CO_3_, the equivalence point was broader, but a small addition of base beyond this still results in overshooting to a maximum above pH 10 (Supplementary Fig. 1), which is outside the confirmed range for stability of the nucleotides. Neutralization with KHCO_3_ also produced a broader equivalence point (Supplementary Fig. 1). More important, even a vast excess of KHCO_3_ resulted in a pH of less than 9. Hence, the nucleotides remain stable even when an excess concentration of base is added. This allows for straightforward neutralization; it is possible to be certain of complete neutralization without the concern of high pH resulting in instability. Therefore, the conditions we use, where the cell extract containing 0.75 M HClO_4_ is neutralized with an equivalent volume of 2 M KHCO_3_, are justified; at no point are conditions produced that induce breakdown of NAADP or conversion of NADP to NAADP.

### Recovery of NAADP during acid extraction

To determine NAADP recovery in our system, we spiked the samples with known amounts of [^32^P]NAADP and then scintillation counted the samples after extraction. The sample was incubated with 251,100 ± 31,424 cpm, and the final neutralized extract contained 162,140 ± 27,191 cpm, yielding a recovery of 65 ± 3.7%. Experiments with unlabeled NAADP show that it is stable during acid extraction and neutralization (see above); therefore, counts in final neutralized sample are likely to be NAADP. Other groups have reported cADPR recovery of 48% using 3 M HClO_4_ [28], 64% using ATP and perchloric acid [29], and 95% using perchloric acid as judged by immunoreactivity [30].

### Synthesis of [^32^P]NAADP

Prior to commencement of the radioreceptor binding assay itself, the appropriate radioligand must be prepared. We routinely synthesize high-specific activity [^32^P]NAADP using a variation of the procedure first reported by Aarhus and coworkers [19]. We have modified the reactions and now commonly get total yields of at least 95% based on total radioactivity.

**Fig. 3:**
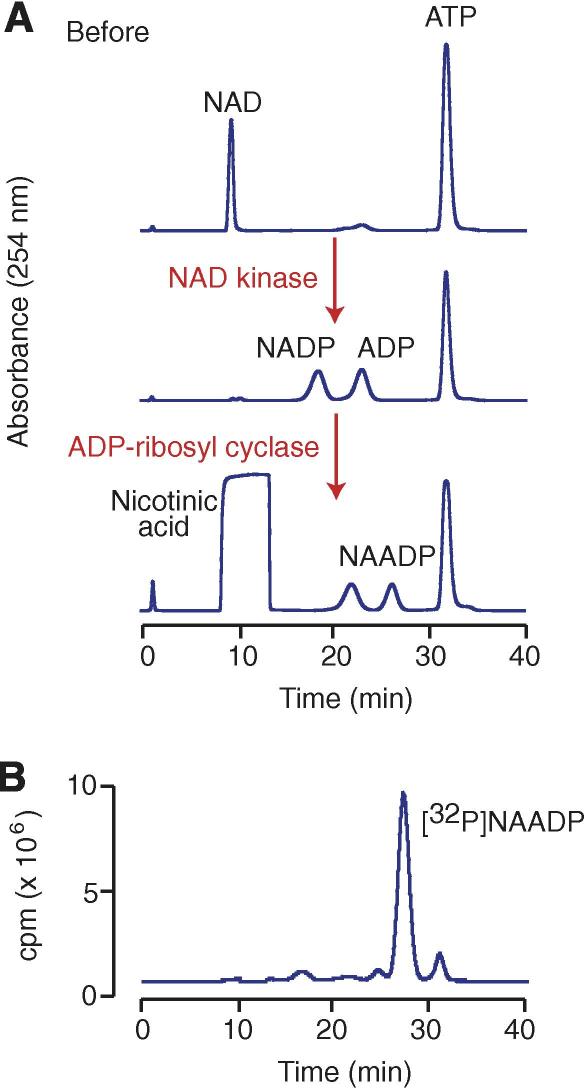
Synthesis of [^32^P]NAADP. (A) HPLC traces of the test reactions using unlabeled compounds. First, NAD is converted to NADP using NAD kinase. Second, NADP is converted to NAADP using ADP-ribosyl cyclase. (B) Separation of the [^32^P]NAADP from the reaction mixture. Detection of unlabeled compounds is performed by UV detection at 254 nm. ^32^P-labeled compounds are detected as counts per minute (cpm) using an in-line Geiger counter.

In the first step of the reaction, the substrates are NAD and ATP. After incubation for 1 h with human NAD kinase, all of the NAD is converted to NADP and an equimolar amount of ATP is converted to ADP (Fig. 3A). Without purifying any intermediates, the reaction is acidified by the addition of nicotinic acid (pH 4) to a final concentration of 100 mM, and Aplysia ADP-ribosyl cyclase is added to a final concentration of 0.1 μg/ml to exchange the nicotinamide for nicotinic acid. After a 1-h incubation, there is complete conversion of the NADP to NAADP (Fig. 3A). Note that the presence of a high amount of nicotinic acid during the HPLC separation alters the elution times of the compounds slightly. This sequence of reactions should be completed each time to confirm enzyme activity.

For the synthesis of radiolabeled NAADP, the entire sequence of reactions is repeated but with smaller volumes. An important consideration was that the human NAD kinase has a high *K*_m_ for NAD (0.8 mM) [26]. Therefore, the volume of the [^32^P]NAD-containing reaction must be minimized to keep the NAD concentration as high as possible. Sea urchin NAD kinase has been used in the past [20], but it must be freshly isolated. Sea urchin egg homogenates, without purification, have been used (data not shown), but both NAD and ATP are metabolized, greatly limiting the efficiency of conversion and the final yield. Human NAD kinase [26] is currently our preferred choice due to the reproducibility and availability. However, judicious choice of substrate concentrations must be made; for example, too much ATP will compete with NAD at its binding site and prevent the reaction. We optimized this conversion by keeping volumes low to maintain a high [^32^P]NAD concentration and chose an intermediate concentration of ATP (5 mM).

Historically, we monitored the elution times of the radioactive compounds by collecting 1-ml fractions and then scintillation counting 1 μl from each fraction in 10 ml of water. An improvement has been implemented at this stage; we now have an in-line radioactivity detector attached to the HPLC. Therefore, we can now collect only the NAADP peak (see Fig. 5B later). This minimizes radiation exposure during the collection, because the fraction collection tray does not need to be moved for each 1-ml sample, but only for the NAADP elution. Typically, the NAADP is collected in two 1.5-ml microcentrifuge tubes. The peak before the [^32^P]NAADP is [^32^P]NAAD (nicotinic acid adenine dinucleotide) (Fig. 3B), synthesized by base exchange of nicotinic acid with the nicotinamide on NAD that was not phosphorylated. This [^32^P]NAAD is the major product in some syntheses if the phosphorylation does not proceed to completion. The small peak after the [^32^P]NAADP is [^32^P]ADPR-P (Fig. 3B) resulting from the base exchange reaction when water is the attacking nucleophile. A very detailed account of this procedure can be found in Morgan and coworkers’ study [18]. A less satisfactory alternative, albeit one that requires less equipment and hence may be considered more financially viable, is to use the [^32^P]NAADP without further purification.

### Determination of sample concentrations using a binding curve

To begin the radioreceptor binding assay, a range of known concentrations of unlabeled NAADP are added to racked test tubes. Sea urchin egg homogenate, containing the binding protein for NAADP, is then added. Samples are incubated for 10 min to allow NAADP to bind irreversibly to its receptor. [^32^P]NAADP is then added, and samples are incubated for a further 10 min to allow [^32^P]NAADP to bind to the remaining free binding protein. Samples are then filtered using a cell harvester. Protein is retained on the filter paper; hence, the total radioactivity bound may be determined (Fig. 1B). Sample concentrations may then be determined from the standard curve.

**Fig. 4:**
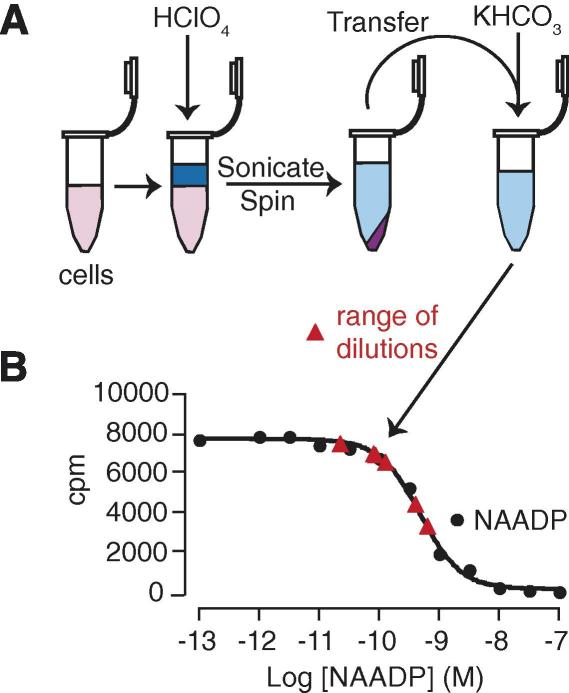
Measurement of NAADP levels in pancreatic acinar cell samples. (A) Schematic diagram showing the preparation of pancreatic acinar cell samples. These samples were then diluted for use in the assay. (B) A competitive displacement curve was generated using standard concentrations of NAADP (black filled circles). Dilutions of the pancreatic acinar cell sample were determined from this curve and are shown on the trace (red triangles). (For interpretation of the references to color in this figure legend, the reader is referred to the Web version of this article.)

To demonstrate that changes in NAADP in cell samples are detected using this method, a cell extract was performed from mouse pancreatic acinar cells. This was then serially diluted to produce a range of more dilute samples. Changes in NAADP were correctly detected from the standard curve (Fig. 4). That is, a given fold dilution resulted in the expected fold change in the NAADP detected.

### Inhibition of binding using related nucleotides and other Ca^2+^-releasing messengers

**Supplementary Figure 2:**
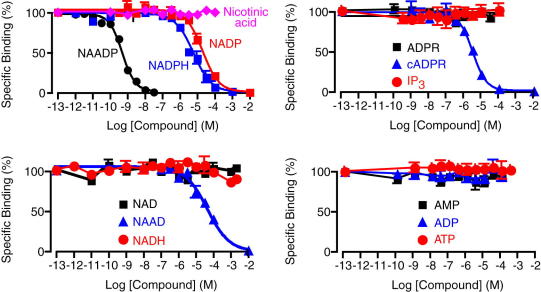


As with all binding assays, a potential problem is selectivity, that is, displacement of [^32^P]NAADP by compounds other than NAADP in the test samples. To examine the extent of this possible problem, we screened several structurally related compounds to establish their relative ability to displace NAADP binding. The full concentration–displacement curves are shown in the supplementary data (see Supplementary Fig. 2). As expected, several compounds compete when present at high enough concentrations. The results can be summarized by the IC_50_ values from the assay as follows: NAADP (540 pM), NADP (21 μM), NADPH (6.3 μM), cADPR (2.5 μM), and NAAD (51 μM). Other compounds failed to displace NAADP at the maximum concentrations tested in the assay: ATP (100 μM), ADP (100 μM), AMP (100 μM), NAD (2 mM), NADH (2 mM), ADPR (100 μM), IP_3_ (100 μM), and nicotinic acid (100 μM). These results are based on commercially obtained compounds (except cADPR, which was synthesized in-house, and NADP, which underwent purification by HPLC). We interpreted these results by noting that all samples result from dilute cell suspensions and are further diluted at least 100-fold in the final assay, and then we calculated the maximum NAADP displacement based on known cellular concentrations of the metabolites. We concluded that none of these compounds presented any worries with regard to interference in the binding assay. For example, ATP has an intracellular concentration of between 1 and 5 mM [31] and would be present in the assay at a maximum of 200 nM; therefore, it would not displace NAADP.

### Validity of assay under cell extract conditions

**Fig. 5:**
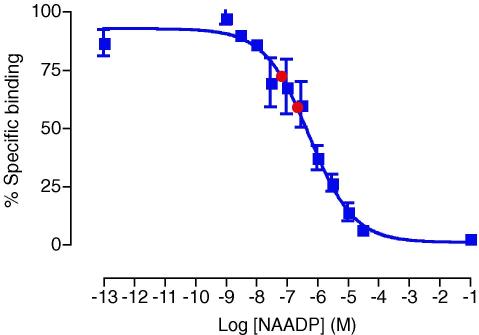
Detection of changes in NAADP concentration under cell extract conditions. The displacement curve was generated from known NAADP concentrations (blue squares). The difference between known NAADP concentrations spiked into rat brain extract (prepared as described in text) is correctly detected (red circles). Error bars represent standard errors of the mean (*n* = 3). (For interpretation of the references to color in this figure legend, the reader is referred to the Web version of this article.)

Although none of the potential inhibitors of NAADP binding tested is able to displace NAADP at the concentrations present in a cell extract, it remains a possibility that the combination of these various compounds present in the cell would be able to displace NAADP and, hence, interfere with the assay. To test this possibility, a sample of chopped rat brain (McIlwain tissue chopper, 650 μm, from 250- to 300-g female Wistar rat) was processed by acid extraction in the manner described in Materials and Methods. This was then spiked with two known 10-fold different concentrations of NAADP. The NAADP in these samples was then analyzed using the radioreceptor binding assay (Fig. 5). The NAADP concentrations determined were 10-fold different, as expected, demonstrating that changes in NAADP are accurately detected under cell extract conditions. Endogenous NAADP prevents precise detection of the original concentrations in this system.

### Improvement of sample detection

**Fig. 6:**
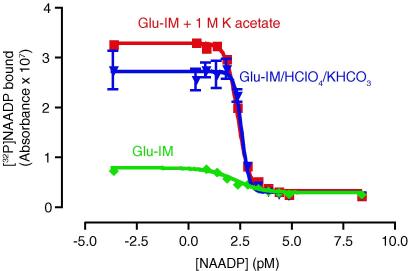
Improvement of NAADP detection in cell extract samples. The green trace shows standards made in intracellular medium (Glu-IM), the blue trace shows standards in “acid-extracted” intracellular medium, and the red trace shows standards in intracellular medium supplemented with 1 M potassium acetate. Error bars represent standard errors of the mean (*n* = 3). (For interpretation of the references to color in this figure legend, the reader is referred to the Web version of this article.)

Initially, standard NAADP concentrations were made up in intracellular medium because this provides suitable conditions for the sea urchin egg homogenate and produces reliable binding curves. However, it was often observed that more radioactivity bound in tubes containing cell extract samples than in the NAADP-free (i.e., intracellular medium-only) standard. Therefore, we decided to make up NAADP in a solution closer to the conditions prevalent in the acid-extracted cell samples. Intracellular medium was mixed with an equal volume of 1.5 M HClO_4_, and this solution was then mixed with an equal volume of 2 M KHCO_3_. The resulting solution was centrifuged to remove the precipitate and then was used to dilute NAADP standard for use in the assay (Fig. 6). The resulting displacement curve has an identical IC_50_ and hill slope to that produced with standards in intracellular medium, but the maximum radioactivity bound increased by approximately threefold. Based on previous reports [23], we hypothesised that the elevated K^+^ in the solution might be responsible for this effect. Therefore, intracellular medium was supplemented with an approximately equivalent concentration of K^+^ in the form of an additional 1 M potassium acetate (pH maintained at 7.4). The binding assay was then repeated with NAADP standards made up in this medium (Fig. 6). The curve produced again had an identical IC_50_ and hill slope, but with a threefold increase in maximum binding. Thus, it is important to use NAADP standards in a buffer with the same K^+^ concentration as the samples to maximize detection, particularly of very small NAADP concentrations.

### Use of storage phosphor screen in place of scintillation counting

**Fig. 7:**
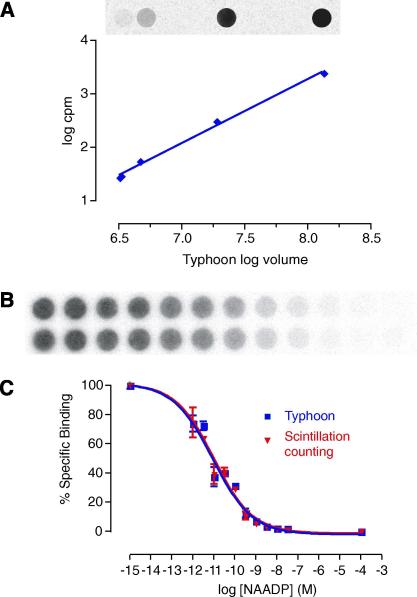
Detection of radioactivity using storage phosphor screens and a Typhoon scanner. (A) Comparison of radioactivity determined by the Typhoon scanner image versus counts per minute (cpm) detected using Cerenkov scintillation counting. (B) Image produced for a standard NAADP displacement curve using the Typhoon scanner . (C) Comparison of standard curves with radioactivity detected using scintillation counting (red symbols and line) and the Typhoon scanner (blue symbols and line). Error bars represent standard errors of the mean (*n* = 3). (For interpretation of the references to color in this figure legend, the reader is referred to the Web version of this article.)

We now describe a number of modifications to the procedure. These modifications aim to reduce exposure to radioactivity, enhance detection of NAADP (particularly in response to agonist stimulation), and enable samples to be analyzed as expediently as possible. Traditionally, radioactivity has been detected using Cerenkov scintillation counting. This method may be used for counting the radioactivity trapped on the filter papers in this assay, and it produces accurate results. However, there are a number of disadvantages to using this technique. Each circle of filter paper must be transferred to an individual vial containing scintillation fluid, and this is a laborious and time-consuming process. Furthermore, disposal of these vials and the scintillation fluid generates a large volume of waste and further exposure to radiation. Therefore, we have adopted a different technique for determining radioactivity in our samples. Whole filter papers (containing 48 samples with our harvester) are wrapped in cling film and then exposed to a storage phosphor screen for 1 to 4 h (depending on the activity of the [^32^P]NAADP). One screen is able to expose to four such filter papers at a time; the times given are suggested minima as the radioactivity decays, but accurate readings may be obtained even after much longer (e.g., overnight) exposures. The storage phosphor screen stores a record of the sample. The screen is then scanned using a Typhoon scanner at a wavelength of 633 nm. As this occurs, blue light (390 nm) is emitted and detected by the scanner. The light intensity is directly proportional to the radioactivity in the sample; in the images, darker pixels relate to a higher light intensity (Fig. 7B).

To confirm that the storage phosphor screen is able to detect the radioactivity in samples over a suitable dynamic range, and to demonstrate that the number of counts could be determined when required, a range of dilutions of [^32^P]NAADP were spotted onto filter papers. These were analyzed first by Typhoon scanning and second by scintillation counting (Fig. 7A). This confirmed that a storage phosphor screen and Typhoon scanning may be accurately used to determine radioactivity in a sample and that it can be calibrated to determine the counts per minute if this information is required. Furthermore, the radioactivity on a filter paper produced in the NAADP radioreceptor binding assay was determined using both techniques (Figs. 7B and 7C). The standard curves generated are identical, further validating the use of this method.

### Measurement of NAADP accumulation over time in agonist-stimulated cells: A novel methodology

**Fig. 8:**
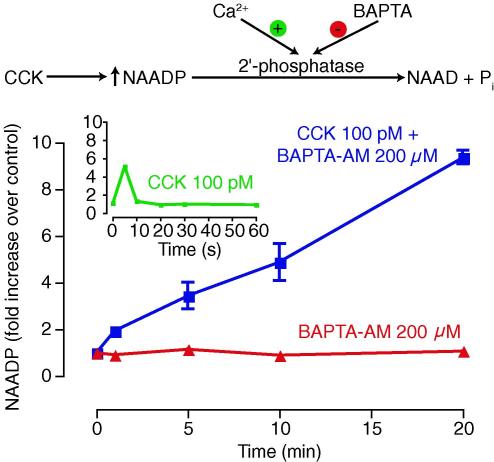
BAPTA-AM, a cell-permeant form of the Ca^2+^ chelator BAPTA, prevents NAADP metabolism and, hence, facilitates NAADP measurements in pancreatic acinar cells. The red trace shows cells preincubated with 20 μM BAPTA-AM, followed by the addition of control buffer and incubations for the various times. The blue trace shows cells that were preincubated with BAPTA-AM, followed by addition of 100 pM CCK for the times indicated. Error bars represent standard errors of the mean (*n* = 3). The inset (green trace) shows an initial peak in NAADP levels in response to CCK in the absence of BAPTA. This response is very transient and, hence, difficult to detect. (For interpretation of the references to color in this figure legend, the reader is referred to the Web version of this article.)

The changes in NAADP levels reported heretofore in mammalian systems consist of a sharp transient early spike in NAADP levels [15], which is easy to miss, especially in an uncharacterized system, depending on the time points chosen. However, NAADP is believed to be metabolized by a 2′-phosphatase that is Ca^2+^ dependent [32]. Therefore, we tested whether the breakdown of NAADP could be inhibited by prior incubation of cells with the cell-permeant form of the Ca^2+^ chelator BAPTA, BAPTA-AM (Fig. 8). Incubation with BAPTA-AM alone produced no change in NAADP levels, but subsequent exposure to CCK produced a continuous increase in NAADP levels that lasted for at least 20 min. Thus, it is possible to detect changes in NAADP in this system by measuring a basal level and then accumulation over time after exposure to agonist. This removes the potential problems of missing a transient increase in NAADP levels because any NAADP synthesized will not be broken down and will remain in the cells for measurement. Thus, the detection of agonists that produce increases in NAADP levels is greatly facilitated, and the problem of “false negatives” is nearly eliminated.

A similar technique has also proved to be useful in measuring changes in the levels of second messengers. For example, metabolism of cAMP may be prevented by the application of methylxanthines [7]. Similarly, changes in IP_3_ may be measured using a radioreceptor binding assay [33]. However, in many systems, including pancreatic acinar cells, the changes are transient and difficult to detect. Therefore, Li^+^ is routinely added to the cells to inhibit metabolism of IP_3_ and, thus, allow measurement of accumulation over time [34].

In conclusion, we have reported and validated a method that allows rapid and accurate measurements of NAADP concentrations. Information was provided in sufficient detail to make the measurement of NAADP accessible to a wider range of laboratories as the field grows. The acid extraction technique discussed is applicable to a wide variety of systems; it may be used in cell cultures, isolated cells, chopped tissue, and indeed whole tissue. The radioreceptor binding assay employed is highly selective for NAADP and is sufficiently sensitive to detect very small concentrations of NAADP. Finally, and most important, we reported a number of improvements to the assay. In particular, the synthesis of [^32^P]NAADP was modified to produce a very high yield with minimal exposure. Furthermore, the detection of radioactivity in samples is now faster, involves less exposure, and produces less waste. In addition, we developed a method to prevent the metabolism of NAADP. This allows the measurement of NAADP accumulation over time, giving more straightforward detection of stimulus-induced increases in NAADP. This development should prove to be very useful in further investigations of the roles of NAADP as a second messenger, just as it has been in the IP_3_ field, and should pave the way for simpler, more sensitive determinations of changes in NAADP levels.

## Footnotes

6 *Abbreviations used:* IP_3_, d-myo-inositol 1,4,5-trisphosphate; NAADP, nicotinic acid adenine dinucleotide phosphate; cADPR, cyclic ADP ribose; NAD, nicotinamide adenine dinucleotide; NADP, nicotinamide adenine dinucleotide phosphate; HPLC, high-performance liquid chromatography; TFA, trifluoroacetic acid; NADPH, reduced-form NADP; ASW, artificial sea water; BAPTA-AM, 1,2-bis(2-aminophenoxy)ethane-*N,N,N′,N′*-tetraacetic acid tetrakis (acetoxymethyl) ester; CCK, cholecystokinin; NADH, reduced-form NAD; NAAD, nicotinic acid adenine dinucleotide.
